# A derived honey bee stock confers resistance to *Varroa destructor* and associated viral transmission

**DOI:** 10.1038/s41598-022-08643-w

**Published:** 2022-04-07

**Authors:** Thomas A. O’Shea-Wheller, Frank D. Rinkevich, Robert G. Danka, Michael Simone-Finstrom, Philip G. Tokarz, Kristen B. Healy

**Affiliations:** 1 Environment and Sustainability Institute, University of Exeter, Penryn, TR109FE Cornwall UK; 2 USDA ARS, Honey Bee Breeding, Genetics and Physiology Laboratory, Baton Rouge, LA 70820 USA; 3 Department of Entomology, Louisiana State University, 404 Life Sciences Building, Baton Rouge, LA 70803 USA

**Keywords:** Agroecology, Ecological epidemiology, Parasite host response, Applied microbiology, Pathogens

## Abstract

The ectoparasite *Varroa destructor* is the greatest threat to managed honey bee (*Apis mellifera*) colonies globally. Despite significant efforts, novel treatments to control the mite and its vectored pathogens have shown limited efficacy, as the host remains naïve. A prospective solution lies in the development of *Varroa*-resistant honey bee stocks, but a paucity of rigorous selection data restricts widespread adoption. Here, we characterise the parasite and viral dynamics of a *Varroa*-resistant honey bee stock, designated ‘Pol-line’, using a large-scale longitudinal study. Results demonstrate markedly reduced *Varroa* levels in this stock, diminished titres of three major viruses (DWV-A, DWV-B, and CBPV), and a two-fold increase in survival. Levels of a fourth virus that is not associated with *Varroa*—BQCV—do not differ between stocks, supporting a disruption of the transmission pathway. Further, we show that when decoupled from the influence of *Varroa* levels, viral titres do not constitute strong independent predictors of colony mortality risk. These findings highlight the need for a reassessment of *Varroa* etiology, and suggest that derived stocks represent a tractable solution to the *Varroa* pandemic.

## Introduction

Honey bee (*Apis mellifera*) colony losses constitute a severe and ubiquitous concern for both the migratory pollination industry, and modern agricultural security in general^1–4^. High annual mortality has become a pervasive facet of commercial pollination, despite significant scientific and legislative efforts to curtail it^5,6^. In the United States alone, current figures place 2018–2019 mortality at 37.5% for commercial beekeeping operations, rising substantially from a 13-year average of 28.8%^7,8^. The causes of this are myriad, and the interrelated nature of extraneous stressors upon colony health makes defining the principle interactions difficult^9,10^. Despite such inherent system complexity, however, the parasitic mite *Varroa destructor* repeatedly emerges as the single greatest driver of global colony losses^1,11–13^.

*Varroa* is associated with varied pathologies, including developmental disruption, immunosuppression, and behavioural alteration^14–16^. An extensive body of research demonstrates that the mite vectors and amplifies a plethora of viral pathogens^17^, several of which are strongly associated with colony mortality^12,18,19^, and the discovery of novel viral types is ongoing^20^. Additionally, recent work has shown that the process of *Varroa* feeding per se may cause significant damage, as these mites consume the honey bee hosts’ fat body—an organ with numerous vital functions^12,21^. Despite containment efforts, *Varroa* has continued its 50-year global expansion into formerly uninfested regions^1,13,22,23^ with concordant implications for managed and wild pollinators^1,24^. Simultaneously, established mite populations show increasing resistance to acaricide treatments, which currently constitute the last effective line of defence^17,25–28^. As such, the ongoing threat presented by this pandemic, and its rapid proliferation through industrialised agricultural systems^29,30^, is both immediate, and severe.

A promising, sustainable solution in the effort to mitigate *Varroa*, lies in the development of *Varroa*-resistant honey bee stocks^17,31,32^. This approach has advantages over other management strategies, in that it is both integrated, and less susceptible to the simple evolutionary adaptations that threaten chemical methods^17,33,34^. In natural *A. mellifera* populations, cases of resistance to *Varroa* mites have been widely reported^35–37^. Due to the devastating nature of infestation absent management, natural selection for colonies able to resist, or coexist, is rapid, and intense^38^. Survival mechanisms are varied, although there is some evidence to indicate convergent trait selection in isolated populations^17,37^. Notably however, many of the mechanisms that predispose colonies to *Varroa*-resistance in nature, such as smaller colony sizes and increased swarming frequencies^38–40^, are antithetical to the characteristics most valued in commercial beekeeping^17^. Indeed, the intensified nature of *Varroa*-host dynamics in managed operations^41,42^, combined with the evolutionary novelty of the association^43^, necessitates resistance mechanisms that are tailored to extreme conditions. Consequently, the selection of heritable resistance traits suited to commercial beekeeping, along with the maintenance of favourable colony-level characteristics, is a significant challenge for breeding efforts^17,38^.

Major programs to develop *Varroa*-resistant commercial stocks are ongoing^38^, with emerging support from marker-assisted selection (MAS)^44,45^, and ‘omics technologies^46^. Artificially selected traits, including *Varroa*-sensitive hygiene (VSH), in which workers remove mite-infested brood^47–49^; low mite population growth (low MPG), which comprises a suite of behaviours limiting *Varroa* reproduction^50,51^; and auto-/allo-grooming, the processes of removing and/or damaging phoretic mites^52,53^, have shown promise in commercial scenarios^17,38,54^. The adoption of resultant lines, however, has been limited^54^, ostensibly due to varying real-world efficacy^31,32,55^, a paucity of controlled large-scale trials with which to validate commercial viability^38,56,57^, and the challenges of scalability in breeding efforts^58,59^. These factors engender industry-wide inertia to the effective integration of *Varroa*-resistant honey bees, and subsequently, the globally managed *A. mellifera* population remains adaptively naïve—and thus acutely susceptible—to *Varroa* parasitism.

Here, we employ a controlled longitudinal analysis, to assess the performance of a novel and genetically distinct *Varroa*-resistant honey bee stock, ‘Pol-line’^54,60^. This stock is unique, in that it possesses the substantial mite-resistance of VSH behaviour^61^, combined with the desirable beekeeping characteristics of commercial Italian colonies; including large population size, substantial honey production, and docile temperament^54^. We utilised a year-long experimental design, comparatively analysing Pol-line and commercial Italian honey bees, in which all colonies were individually tracked, and parasite, pathogen, and health measures taken at key time points. Colonies were integrated into a migratory operation in the USA, focussing on almond pollination and honey production, and spanning the states of Mississippi, South Dakota, and California. This system is notable, as it constitutes one of the most intensive and stressful scenarios in modern beekeeping^62,63^, while simultaneously incorporating three distinct Köppen–Geiger climate zones^64^. As such, it provides a rigorous test of the functionality of colonies, and is not limited to localised applicability. The study design manipulated acaricide application, migration route, and colony management, to compare the performance of both stocks, while monitoring *Varroa* levels, and associated viral pathogen titres, with high fidelity.

In addition to assessing the functionality of Pol-line honey bees, we utilised our experimental system to examine the relative influences of *Varroa* and its associated viruses upon mortality in the field, along with the predictive utility of both for informing colony prognoses. While the influence of *Varroa* upon viral dissemination and amplification has been investigated extensively^10,17^, the specific impacts of *Varroa* feeding per se*,* versus those of the viruses that it transmits, remain poorly understood at the bipartite level^12,17,29^. This paucity of information arises from the difficulty of isolating either factor effectively, given their tightly coupled nature, and the requisite cost of controlled field studies^1,12,65–67^.

Current understanding places the RNA picornavirus Deformed wing virus (DWV)—specifically the master variants DWV-A^68^, and DWV-B^69^—as the prime viral threat to colony health^70^. Impetus for this is derived from the correlation that DWV exhibits with colony mortality, and the disseminative properties of the virus when vectored by *Varroa*^23,71^. In the presence of *Varroa* infestation, DWV can increase from latent levels of 6–13%, up to 100% prevalence in colonies, accompanied by a million-fold increase in viral titres within 3 years^1,22^. In tandem, there is evidence for the recombination of DWV strains, leading to more virulent hybrid forms, and crucially, selection for these forms when transmitted as a viral admixture, via *Varroa* feeding^68,70,72^. While the prevalence of DWV is well established, investigations of the role of viral diversity and propagation in determining pathogenicity have shown inconsistent results^68,73–78^, and surprisingly, some studies linking the virus to mortality do not account for the effect of *Varroa* levels on colony outcome^73,79–81^. The latter may be symptomatic of conceptualising *Varroa* as a viral vector first, and damaging agent second; however this view is inconsistent with both the mite’s biology^12,17^, and established large-scale datasets^67,76,82,83^.

Aside from the known *Varroa*-vectored viruses, certain other pathogens exhibit *Varroa* association, although their exact relationship with the mites is unclear^17^. One such case is the taxonomically unassigned RNA virus, Chronic bee paralysis virus (CBPV)^84,85^. Despite being one of the first described honey bee viruses^86^, CBPV is comparatively under-studied. However, it is known to cause mortality in adult bees^84^, to correlate with colony losses^87–89^, and has a wide host range^86,90^, with an increasing global incidence^86,91^. Notably, CBPV can exist at covert levels in healthy colonies, may increase in prevalence in the presence of *Varroa* parasitism^92–94^, and has been detected in *Varroa*^90,95^. A direct transmission pathway remains elusive, however^84^, and observational studies suggest variable association^90,96^.

Another potential *Varroa* associate is the RNA picornavirus Black queen cell virus (BQCV)^17,97,98^. BQCV is highly prevalent globally^97^, causes fatal infections in queen larvae^99^, and yet generally remains asymptomatic in adult workers^98,99^, showing limited association with colony losses^100,101^. As with CBPV, BQCV has been detected in *Varroa*^95,102^, and there is evidence for increased transmission to other hosts when the mites are present^103^. Notably however, while some studies have found that BQCV titres are influenced by *Varroa* infestation^22,104^, others indicate that the virus is decoupled from *Varroa* levels in colonies^105–107^, and conclusive proof of *Varroa* transmission is yet to be ascertained^22^. The former point is salient, as current understanding suggests that although *Varroa*-mediated transmission of BQCV may be feasible, its occurrence is, at most, rare^98^. Hence, quantifying this somewhat cryptic relationship will be of utility to the wider study of *Varroa*-virus dynamics.

Conflicting findings from etiological studies of DWV^66,73,76,81,108^, CBPV^90,94,106,109^, and BQCV^17,95,105^, exacerbated by a historically inaccurate research paradigm for *Varroa* feeding, which appears to have underestimated direct damage to the fat body^12^, exemplify the need for ongoing characterisation of *Varroa*-virus mediated colony mortality. Until this process is better understood, it remains an obstacle to effective treatment, because there is no clear consensus as to the relative importance of covariate factors^1,12,66,110,111^.

Thus, using the longitudinally tracked colonies in our experimental setup, we tested the influence of *Varroa* levels, and DWV-A, DWV-B, CBPV, and BQCV titres, on colony mortality. This constituted an assessment of viruses both tightly (DWV-A and DWV-B), and loosely (CBPV and BQCV), linked to *Varroa* parasitism. We then controlled for *Varroa* levels, grouping colonies by mite infestation strata, to characterise the *relative additive* predictive power of each virus in determining colony mortality, using an epidemiological approach. As such, we aimed to evaluate the performance of a novel *Varroa*-resistant honey bee stock, while simultaneously investigating the specific factor weightings of *Varroa*-mediated colony mortality.

## Materials and methods

### Colonies

Colony setup occurred prior to initiation of the study, between March and May 2017, in Mississippi, USA. Using established methods, queenless colony divisions, obtained from a large commercial beekeeping operation, were equalised to an average calculated population size of ~ 7000 workers^112^, and housed in 10-frame Langstroth hives (Table S1). After acclimatisation for 24–48 h, they each received an imminently emerging queen cell, containing a queen from one of two stocks, added to the same worker baseline. The stocks used consisted of an Italian ‘Commercial’ stock, propagated from collaborator established breeder queens, and thus representative of the industry standard, and the *Varroa*-resistant ‘Pol-line’ stock^54^. To ensure consistency, all queens were reared in the same ‘cell builder’ colonies, based at the USDA Honey Bee Breeding, Genetics and Physiology Laboratory, in Baton Rouge, Louisiana, USA. Colonies from each stock were held in independent apiaries, 80 km apart to maintain physical isolation; and to control genetic fidelity, virgin queens were open mated to drones of the same stock via drone saturation. Fourteen days after queen emergence, colonies were inspected, and mated queens were marked with paint on the thorax, to assist with identification, with white corresponding to Commercial, and blue to Pol-line. Colonies were allowed to acclimatise for six weeks before sampling began, and those that failed to achieve mating success, or had unacceptably high [≥ 3.0 ‘mites per hundred bees’ (MPHB)] *Varroa* levels, were removed, normalising the average between-stock *Varroa* difference to < 0.1 MPHB from the initiation of the study (Table S1). Each colony was then assigned a random ID number, for a total of 366 colonies; 193 with Commercial queens, and 173 with Pol-line queens. All colonies were provided with supplemental sucrose solution, and soya-based commercial pollen supplements ad libitum, in accordance with standard industry practices.

### Experimental design

**Figure 1:**
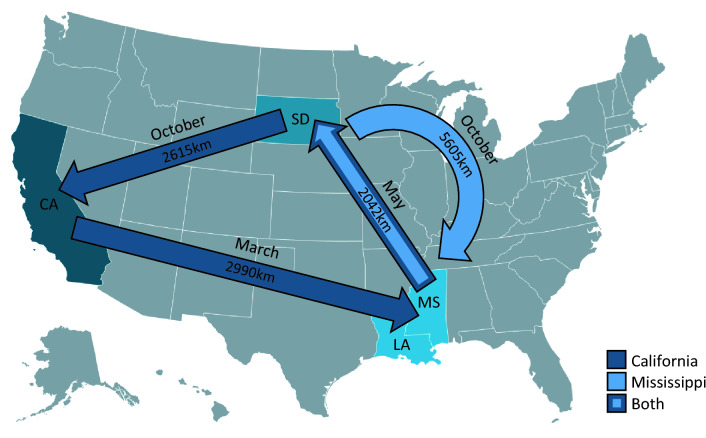
Migration routes used in the experimental setup. Arrows indicate travel routes, distances, and timings for each migration group (California, dark blue; Mississippi, light blue; both, dark and light blue). Choropleth map generated using Datawrapper (release v. 1.25.0).

In order to evaluate the effects of migratory pollination procedures, colonies were randomly assigned to one of three migration route treatments. The migration treatments used were as follows; a ‘California’ group, consisting of 156 colonies; 80 Commercial and 76 Pol-line, moved to South Dakota in May, followed by California in October for overwintering and almond pollination, and back to Mississippi at the end of the study; a ‘Mississippi’ group, consisting of 156 colonies; 80 Commercial and 76 Pol-line, moved to South Dakota in May, and back to Mississippi via California in October for overwintering; and a ‘Stationary’ group, consisting of 54 colonies; 32 Commercial and 22 Pol-line, that remained in Mississippi for the duration of the study (Fig. 1). While in South Dakota, colonies were distributed across four apiaries, and in California and Mississippi, they were held in a single apiary. This practice mirrored standard industry protocol, and provided a more representative sample by accounting for localised differences in climate and forage.

In addition to migration route, colonies were divided by the frequency of acaricide treatment applied, to better elucidate the effects of varying infestation strata. Within each migration treatment, and stock, half of the colonies received a ‘high’ acaricide treatment, and half a ‘low’ acaricide treatment. Colonies in the high treatment group were treated twice, in September and December; while those in the low treatment group were treated only once, in December. All treatments were conducted using the acaricide amitraz.

### Sampling

Colonies were sampled five times during the course of the study, in May, June, September, December, and February. For each colony, the queen was examined, and frames of workers, nectar, pollen, and brood were quantified visually by percentage cover^113^. Upon observation, if the queen did not have an existing mark, a single red paint mark was applied to her thorax, to indicate supersedure status. Then, ensuring that the queen was sequestered to prevent unintentional sampling, two samples of ~ 300 workers were taken in resealable bags; one being flash frozen with dry ice, and stored at − 80 °C for pathogen analyses; and the other being frozen with conventional ice, for *Varroa* analysis. To achieve a representative age sample, workers were removed from two brood frames, containing sealed and unsealed brood. Workers were evenly mixed using a 20 L bucket, before collection via a cup-measure, and subsequent assignation to one of the two sample types. The queen was then returned to the hive, along with any unsampled workers.

### *Varroa* analysis

*Varroa* analyses were conducted for all colonies, at all five time points. To quantify the level of *Varroa* infestation in colonies, a detergent-wash method was employed^114^. The sample of bees was first placed in a mesh-partitioned cup with a solid base, to allow mite retrieval. Water was then added until the bees were submerged, and a surfactant-based detergent (Procter & Gamble) mixed in, ensuring an even coating. The lid of the cup was secured, and it was placed into a Model E5850 reciprocal shaker (Eberbach), to be agitated for a period of 60 min at 120 rpm. After agitation, the lower faction of the cup was removed, and the contents poured into a shallow tray, to count the number of mites falling through the mesh. This process was repeated until two consecutive zero counts were recorded, indicating that all mites had been removed. The bees were then separated and counted by tally counter, to produce an exact sample size. Finally, the total *Varroa* count was transformed to produce a standardised ‘mites per hundred bees’ (MPHB) value, as follows:

$${\text{MPHB}} = \left( {{\text{total mites}}/{\text{total bees}}} \right) \times {1}00$$

### Pathogen analyses

Four specific viral targets were chosen: DWV-A, DWV-B, CBPV, and BQCV. Analyses were carried out on a randomised subset of 92 colonies from the California and Mississippi migration groups, divided evenly across stock, and survival outcome. Four major time points were selected: June, September, December, and February. All analyses were conducted using RT-qPCR, following previously established methods^94,115^.

### RNA extraction

Per colony and time point, pools of 65 bees were randomly selected from the master samples of ~ 300, placed into 30 ml 19-6358Z bead tubes (Omni), and stored at − 80 °C, for homogenisation using a Bead Ruptor Elite (Omni). Immediately after homogenisation, 5 ml of homogenisation solution (Promega), and 4 ml sterile 1xPBS buffer, each at 4 °C, were added to sample tubes, before vortexing for 20 s using a Vortex-Genie 2 (Scientific Industries). 1.8 ml of each sample was then transferred to 2 ml tubes (Eppendorf), and centrifuged for 60 s at 5000 rpm, at 4 °C, using a 5430-R centrifuge (Eppendorf). Per sample, 400 μl of the resultant supernatant was then transferred to Maxwell RSC-48 cartridges (Promega), prepared in accordance with the manufacturer’s instructions, and RNA extractions were carried out using the Maxwell RSC simplyRNA tissue protocol (Promega) (Maxwell RSC simplyRNA Tissue Kit, AS1340, TM416, 2019).

Sample purity and yield were then calculated in duplicate, using a NanoDrop One microvolume UV–Vis spectrophotometer (Thermo Fisher Scientific), and appropriate RNA dilutions were completed to ensure sample standardisation to 100 ng/μl. Following standardisation, cDNA synthesis was achieved using QuantiTect Reverse Transcription Kits (Qiagen), utilising a T100 thermal cycler (Bio-Rad), running custom QuantiTect cDNA synthesis protocols (Qiagen) (QuantiTect Reverse Transcription Handbook, 2009). Resultant samples were stored at − 20 °C, prior to initiation of RT-qPCR analyses.

### RT-qPCR

RT-qPCR was performed on 1 μl aliquots of each sample, in triplicate, in a total reaction volume of 10 μl, utilising SsoAdvanced Universal SYBR Green Supermix (Bio-Rad), on Multiplate 96-well optical PCR plates (Bio-Rad). The primers used for quantification of DWV-A^116^, DWV-B^117^, CBPV^96^, and BQCV^115^ have been reported previously, and validated for target specificity. For full sequence details, see (Table S2). Negative controls were included for each target, consisting of 1 μl of nuclease-free H_2_O (Promega), again run in triplicate. Additionally, to enable viral quantity mean determination, standard curves were produced for all targets, via tenfold serial dilutions of known viral quantities, covering 8 orders of magnitude. Linearity (r^2^), and reaction efficiency (E), were maintained at ≥ 0.990, and ≥ 92.5% respectively, for all assays (Table S2). All analyses were run on CFX Connect Real-Time PCR Detection Systems (Bio-Rad), using previously optimised thermal protocols (Table S3).

### Data transformation

Samples were analysed in triplicate, to form a mean Ct value. Any triplicates with a standard deviation ≥ 1.0 were examined, and the divergent replicate removed, resulting in 26 technical replicate removals. If this failed to bring the standard deviation to < 1.0, the sample itself was replaced, and removed from further analyses. Ct means were then quantified against the standard curves for each target, to calculate absolute viral titres, using the following equation:

$${\text{Quantity mean}} = {1}0^{{(\left( {{\text{standard curve y - intercept }} \times {\text{ Ct mean}}} \right) + ({\text{standard curve x - intercept}}))}}$$

Due to the wide distribution of quantities inherent to viral replication dynamics^118^, these values were then log-transformed, to produce suitable data for further analyses. Where undetected values were present, a constant Ct mean of 45 was assumed, to allow for subsequent transformation of the data^105^.

### Range of assessment factors

Analyses were broadly divided into measurements of colony survival, the extraneous influences of *Varroa* levels (MPHB), and viral titres (log_10_ viral quantity mean per μl cDNA) upon them, and how stock, mite treatment, and migration route modulated these interactions. Crucially, we examined both the general effect of *Varroa* levels and viral titres upon colony survival, and the predictive power of such measures at seasonally-relevant time points. We additionally determined the explanatory power of viral titres when colonies were matched for *Varroa* level, thus elucidating the relative additive influences of *Varroa*-transmissible viruses.

#### Colony and queen survival over time, population size, and honey production

First, the survival of colonies and queens over time, along with the population sizes of surviving colonies, and honey production, were compared between the two stocks using time series data collected throughout the course of the study. Colonies were classified as ‘dead’ when the worker frame-count dropped to < 1.0, and they were confirmed as vacant during any sequential sampling. This definition of colony death remained consistent across all further analyses. Queens were classified as ‘dead’ when the existing queen could not be found, and a new unmarked queen, or queen cells, were present, indicating supersedure^119^. Population sizes were compared using worker frame-counts from all colonies classified as ‘alive’, that is, those with ≥ 1.0 frames at the end of the study, in February. Honey production was quantified via net weight (kg) of honey extracted per colony during September, excluding colonies in the stationary migration group, for which extraction was not recorded.

#### Factors influencing colony survival

We then defined the influences of *Varroa* levels and viral titres upon colony survival, using data from all five and four time points, respectively. The effect of each factor, along with stock, mite treatment, migration route, and their concordant interactions, was quantified. By tracking individual colonies over time, in concert with their *Varroa* levels, and viral titres, we were able to determine factor effect sizes, and the interactions leading to death or survival in February. February was chosen as the time point of interest, as it fell immediately prior to almond pollination, and thus gave a representative, and commercially relevant, measure of colony strength going into this system. As viral titre measures were taken for a subset of colonies, separate analyses were conducted for these and *Varroa* measures, in order to preserve maximum sample sizes in each case. These data were then used to inform subsequent predictive analyses, based on *Varroa* levels and viral titres.

#### Predictive differences in Varroa levels and viral titres

To assess initial predictive differences in *Varroa* levels and viral titres, across survival outcomes and stocks, we linked survival status in February to two predictive time points: June, and September. Notably, the former of these encompassed the principle period of colony population growth, and the latter, of honey harvesting, prior to colony overwintering. These points constitute key junctures at which management decisions must be made, and thus are practically relevant as predictive benchmarks. In analyses, colonies were classified by stock, or their status as being either alive or dead in February. *Varroa* levels and viral titres were then compared between groups, thus representing a basic test of the usefulness of single time point data in defining outcomes.

#### Prognostic power of Varroa levels and viral titres

To quantify the epidemiological significance of *Varroa* levels and viral titres in determining colony survival, and therefore their predictive power, we conducted relative risk (RR) analyses for these factors, utilising colony death by February as the outcome of choice. RR is an established epidemiological measure, used to determine the probability of a given health outcome when exposed to a specific risk factor, and thus compare risk magnitudes^120^. RR analyses were conducted for both *Varroa* levels, and viral titres, again for the predictive time points of June and September. Significant interactions were further validated with attributable risk (AR) analyses, to provide a measure of effect size; and complementary Bayesian RR analyses, to visualise the risk probability distribution. Analyses were pooled across stocks, in order to better encompass the full range of infestation and infection strata. Additionally, they were stratified by *Varroa* level, to determine how changes in infestation severity influenced the risk of colony death.

We then isolated the additive effect of viral titres as a predictive factor, independent of *Varroa*. We considered this an important test of the stand-alone influence of *Varroa*-associated pathogens in *Varroa*-mediated colony loss, as ordinarily, these factors are difficult to decouple. To achieve this, a subset of 60 colonies were matched by September *Varroa* level (mean MPHB, < ± 0.1 between survival outcomes), and left unstratified in terms of viral titres. September was chosen as the key matching point for this analysis, as colonies showed the highest *Varroa* levels and viral titres at this stage, and thus these values were considered most relevant to overwinter survival outcomes. Colonies were subsequently divided into alive and dead cohorts, based on their status in February, to produce groups that differed in their survival outcomes, and viral titres, but that had tightly coupled *Varroa* level distributions. We then conducted RR analyses for this subset, examining viral titres in both June and September as predictive factors for colony survival, hence constituting a measure of viral effects, decoupled from *Varroa*.

#### Varroa model

To demonstrate the relationship between *Varroa* level and colony mortality, and generate a predictive model for treatment prognoses in both stocks, we pooled all colony data into *Varroa* infestation strata, derived from *Varroa* level in September, and assigned resultant percentage mortalities based on survival rates in each stratum. We then fit curve models to the data for the two stocks, thus enabling a predictive mortality outcome for the *Varroa* level and stock in question, based on an extensive empirical dataset.

### Statistical analyses

For all pairwise comparison data in which analyses would assume a normal distribution, we performed Shapiro–Wilk tests to check for normality, and hence inform the application of appropriate statistical tests. In all cases, the data were not normally distributed, and thus independent sample Mann–Whitney U-tests were employed. For these analyses, we opted to report mean ranks, rather than medians, as based on descriptive statistics, we did not assume identical frequency distributions between sample groups. Due to the large sample sizes used, we also considered it important to include a measure of effect size, and this was achieved via Eta-squared (η^2^) post hoc tests^121^. Additionally, for analyses that were close to the threshold of significance (0.05–0.06), we cross-verified results by applying parametric methods to transformed data. This occurred in one case, and after transformation via reflection of the square root, the significance of the test was unaltered.

Cumulative colony and queen survival differences between stocks were assessed using mixed-effects Cox proportional hazards models, via the R packages ‘coxme’^122^ and ‘multcomp’^123^. The proportional hazards assumption was verified in each case by confirming independence between the Schoenfeld residuals and time, for both the fixed factors and model, using the R package ‘survival’^124^.

To assess differences between stocks in colony population size and honey production, we utilised generalised linear models (GLMs). The population size model used February as the response time point, while the honey production model used September. Model selection was based on AIC, and validated using omnibus tests, along with assessment of the deviance to degrees of freedom ratios.

To determine how *Varroa* levels and viral titres influenced colony survival outcome, and how stock, mite treatment, and migration route modulated these interactions, we employed generalised linear mixed models (GLMMs) with repeated measures. Separate models were generated for *Varroa* level and viral titre analyses, in order to maximise sample sizes, and minimise model complexity. *Varroa* level measurements were repeated over five time points, and viral titre measurements over four. Model selection was based on AIC, beginning with the full model and interactions. In all cases, model fit was validated via evaluation of the binned standardised residuals.

When testing the predictive power of *Varroa* levels and viral titres at single time points, we used relative risk (RR) analyses. All RR analyses made use of an ‘outgroup’ for the epidemiological factor in question, and then compared the mortality rate in this group to that of an ‘exposure’ group, to ascertain the relative change in risk. The exposure group in all viral titre analyses was defined as colonies with titres ≥ 1 × 10^7^, a cut-off value indicative of an epidemiologically severe infection^1,96,98^, while the outgroup was composed of colonies with titres < 1 × 10^7^. The exposure groups in the *Varroa* level analyses were stratified to cover the range of MPHB levels present in colonies, ≥ 1–2.5, > 2.5–5, > 5–7.5, and > 7.5, and the outgroup was maintained at < 1, thus providing a graded measure of the change in risk with increasingly severe infestations. RR values greater than one indicated an associated increase in mortality risk, while those less than one indicated an associated reduction. In accordance with standard interpretation, RR values were deemed not significant if the resultant 95% confidence intervals overlapped the value of one^120^. For significant RR values, we also calculated the AR, to demonstrate the magnitude of absolute effect size, as is recommended for binary epidemiological outcomes^125^. Significant risk probability distributions were then visualised via parallel Bayesian RR analyses, using the R package ‘brr’^126^. Across tests, we confirmed requisite sample sizes to provide a minimum power (1 − β) of 0.80, at an alpha (α) of 0.05, using standard deviation (σ) and mean difference (δ) values from the data. All statistical analyses were performed in PASS (release v. 21.0.2), SPSS (release v. 25.0.0.0), and R (release v. 4.0.2)^127^.

#### Colony and queen survival over time

We used mixed-effects Cox proportional hazards models to assess differences in colony and queen survival between the two stocks over the course of the study. These utilised stock, mite treatment, and migration route as fixed factor predictors, and colony ID as a random factor. As sampling occurred every two months, cumulative survival was updated on a bi-monthly basis, over a 10 month period.

#### Population size and honey production

The GLM assessing the effect of stock on colony population size used frame-count of surviving colonies in February as a gamma response variable with a log link, and stock, mite treatment, and migration route as fixed factor predictors. The GLM assessing the effect of stock on honey production used net honey weight in September as a gamma response variable with a log link, stock and mite treatment as fixed factor predictors, and omitted migration route as honey was extracted prior to transport.

#### Factors influencing colony survival

The GLMM assessing the effect of *Varroa* levels on colony survival used colony survival status in February as a binomial response variable with a probit link, *Varroa* MPHB, stock, mite treatment, and migration route as fixed factor predictors, their two-way interactions, and colony ID as a random factor. The GLMM assessing the effect of viral titres on colony survival used colony survival status in February as a binomial response variable with a logit link, log_10_ DWV-A titre, log_10_ DWV-B titre, log_10_ CBPV titre, log_10_ BQCV titre, stock, mite treatment, and migration route as fixed factor predictors, their two-way interactions, and colony ID as a random factor.

#### Predictive differences in Varroa levels and viral titres

Independent sample Mann–Whitney U-tests were utilised to compare differences in mean *Varroa* levels and viral titres, based on colony survival outcome and stock, for June and September time points.

#### Prognostic power of Varroa levels and viral titres

To quantify the relative predictive powers of *Varroa* levels and viral titres in June and September when determining colony mortality, and that of viral titres when matched for *Varroa* level, we used RR, and AR, analyses. To visualise risk probability distributions, we used Bayesian RR analyses, utilising a Poisson response, and the Jeffreys prior as an objective reference prior.

#### Varroa model

A curve estimation analysis was employed to fit curve equations to the *Varroa* model data, and curve selection was evaluated via the resultant model outputs, based on F-values.

## Results

### Colony and queen survival over time

**Figure 2:**
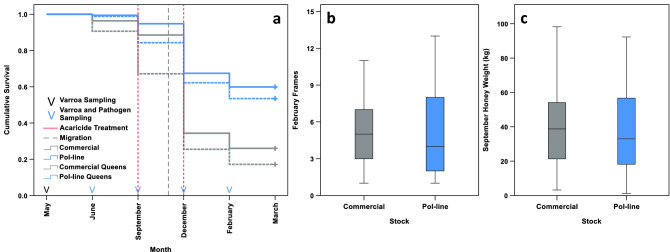
(**a**) Survival proportions of colonies (solid lines), and queens (dashed lines), of each stock (Commercial, grey; Pol-line, blue), across the course of the study (*N*_Commercial_ = 193, *N*_Pol-line_ = 173). Dashed vertical lines indicate the timings of management events (acaricide treatment, red; migration for overwintering, grey). Arrows designate the timings of sampling (*Varroa* sampling, black; *Varroa* and pathogen sampling, blue). Significant differences between stocks were present in colony survival (hazard ratio = 0.408, χ^2^ = 122.800, d.f. = 1, *N* = 366, *P* < 0.001), and queen survival (hazard ratio = 0.355, χ^2^ = 234.388, d.f. = 1, *N* = 366, *P* < 0.001). (**b**) Frames of adult bees in surviving colonies in February (*N*_Commercial_ = 50, *N*_Pol-line_ = 103). Frame counts did not differ significantly between the two stocks (*χ*^*2*^_1_ = 2.272, *N* = 153, *P* = 0.131). (**c**) Honey production in September (*N*_Commercial_ = 141, *N*_Pol-line_ = 137). Honey production did not differ significantly between stocks (*χ*^*2*^_1_ = 0.476, *N* = 278, *P* = 0.490).

Percentage survival over time was significantly greater in Pol-line colonies when compared to Commercial colonies (Mixed-effects Cox proportional hazards model, effect of stock: hazard ratio = 0.408, *χ*^2^ = 122.800, d.f. = 1, *N* = 366, *P* < 0.001, percentage survival_Pol-line_ = 59.884%, percentage survival_Commercial_ = 26.042%; Fig. 2a), and in colonies receiving the high mite treatment, as opposed to the low mite treatment (Mixed-effects Cox proportional hazards model, effect of mite treatment: hazard ratio = 0.516, *χ*^2^ = 25.192, d.f. = 1, *N* = 366, *P* < 0.001, percentage survival_high_ = 57.051%, percentage survival_low_ = 30.769%). Additionally, cumulative survival differed significantly between migration treatments (Mixed-effects Cox proportional hazards model, effect of migration route: *χ*^2^ = 10.854, d.f. = 2, *N* = 366, *P* = 0.004, percentage survival_Stationary_ = 26.415%, percentage survival_California_ = 39.355%, percentage survival_Mississippi_ = 50.000%). This difference was explained by higher survival in the California (Mixed-effects Cox proportional hazards model, Benjamini–Hochberg post hoc test: hazard ratio = 0.607, *z* = − 2.854, d.f. = 2, *P* = 0.004, percentage survival_California_ = 39.355%, percentage survival_Stationary_ = 26.415%), and Mississippi (Mixed-effects Cox proportional hazards model, Benjamini–Hochberg post hoc test: hazard ratio = 0.570, *z* = − 3.206, d.f. = 2, *P* = 0.002, percentage survival_Mississippi_ = 50.000%, percentage survival_Stationary_ = 26.415%) migration groups, when compared to the Stationary migration group. Colony ID had a significant random effect (colony random effect: variance = 0.393, P < 0.001).

Percentage queen survival over time was also significantly greater in Pol-line colonies when compared to Commercial colonies (Mixed-effects Cox proportional hazards model, effect of stock: hazard ratio = 0.355, *χ*^2^ = 234.388, d.f. = 1, *N* = 366, *P* < 0.001, percentage queen retention_Pol-line_ = 88.350%, percentage queen retention_Commercial_ = 66.000%; Fig. 2a), marginally independent of mite treatment (Mixed-effects Cox proportional hazards model, effect of mite treatment: *χ*^2^ = 3.603, d.f. = 1, *N* = 366, *P* = 0.058), and migration route (Mixed-effects Cox proportional hazards model, effect of migration route: *χ*^2^ = 6.857, d.f. = 2, *N* = 366, *P* = 0.052). Colony ID again had a significant random effect (colony random effect: variance = 0.852, P < 0.001).

### Population size and honey production

The population size of surviving colonies in February did not differ significantly between the two stocks (GLM, effect of stock: *χ*^2^_1_ = 2.272, *N* = 153, *P* = 0.131; Fig. 2b), or mite treatments (GLM, effect of mite treatment: *χ*^2^_1_ = 0.735, *N* = 153, *P* = 0.391), with migration route being the only significant factor (GLM, effect of migration route: *χ*^2^_2_ = 8.935, *N* = 153, *P* = 0.011, mean frame-count_Stationary_ = 3.240, mean frame-count_Mississippi_ = 4.597, mean frame-count_California_ = 5.614). Specifically, this difference was accounted for by higher frame counts in surviving colonies within the California migration group, when compared to those in the Stationary migration group (GLM, Holm-Bonferroni post hoc test: *t* = 3.154, d.f. = 1, *P* = 0.005, mean frame-count_California_ = 5.614, mean frame-count_Stationary_ = 3.240). Similarly, honey production did not significantly differ between stocks (GLM, effect of stock: *χ*^2^_1_ = 0.476, *N* = 278, *P* = 0.490; Fig. 2c), or mite treatments (GLM, effect of mite treatment: *χ*^2^_1_ = 0.371, *N* = 278, *P* = 0.542).

### Factors influencing colony survival

**Figure 3:**
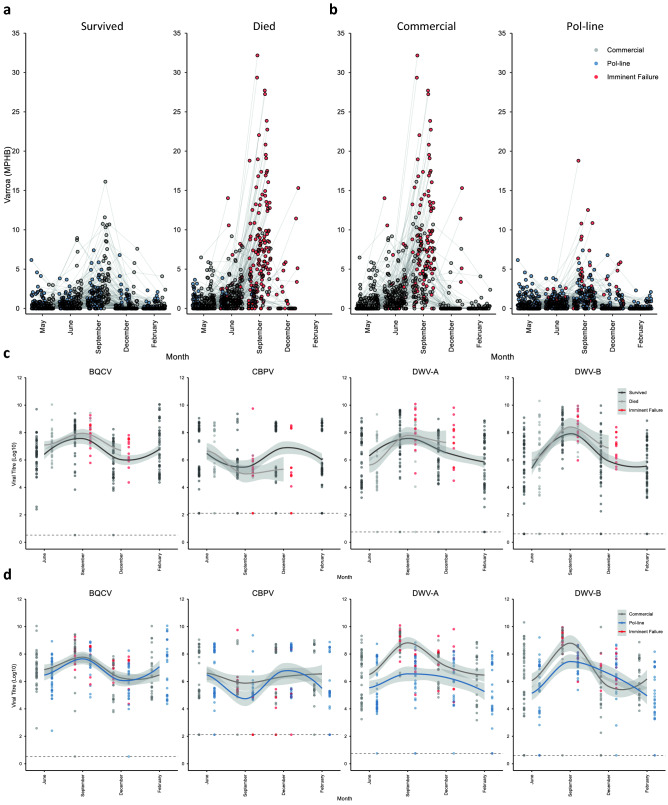
(**a**,**b**) *Varroa* levels in colonies, divided by survival outcome (**a**), and stock (**b**), across the course of the study (*N*_Commercial_ = 193, *N*_Pol-line_ = 173). Each point represents a single measure of *Varroa* for the corresponding month, and lines link repeated measures for the same colonies over time. Points are coloured based on stock (Commercial, grey; Pol-line, blue), and colonies that died by the following sampling point are highlighted (imminent failure, red). This latter designation is included to visualise the *Varroa* levels of colonies prior to failure. *Varroa* level had a significant influence upon colony survival outcomes (*F*_1,373_ = 53.124, *P* < 0.001). (**c**,**d**) Viral titres in colonies for each survival outcome (**c**), and stock (**d**), divided by target, across the course of the study (*N*_Commercial_ = 46, *N*_Pol-line_ = 42). Points represent single month measures of viral titre for colonies, offset at each time point by survival outcome in February (survived, dark grey; died, light grey) (**c**), or stock (Commercial, grey; Pol-line, blue) (**d**). Colonies that were dead by the following sampling point are highlighted (imminent failure, red), and offset to their concordant group. This designation is included to visualise the viral titres of colonies prior to failure. Solid lines indicate LOESS (locally weighted scatterplot smoothing) moving averages, coloured according to their corresponding group. Shaded areas represent 95% confidence intervals. Dashed lines designate the baseline Ct detection limits for each target, after transformation. Viral tires of DWV-A (*F*_1,66_ = 1.026, *P* = 0.315), DWV-B (*F*_1,85_ = 0.206, *P* = 0.651), CBPV (*F*_1,80_ = 0.516, *P* = 0.475), and BQCV (*F*_1,69_ = 3.142, *P* = 0.081), did not have significant effects upon colony survival outcomes.

When examining the effect of *Varroa* level, survival status in February was significantly influenced by colony stock, mite treatment, migration route, and *Varroa* level (Fig. 3a,b) (Table 1). Additionally, significant interactions occurred between stock and mite treatment, and stock and *Varroa* level, indicating the importance of stock as a modulating factor (Fig. 3b; Table 1). The interaction between mite treatment and *Varroa* level was not significant (Table 1), suggesting that although *Varroa* level was reduced by mite treatment, this did not alter its underlying relationship with colony survival.

**Table 1:** Summary statistics for GLMM assessing the effect of *Varroa* levels on colony survival.

Type	Factor	Statistic	d.f	*P*
Fixed effect	Stock	*F* = 49.233	1, 371	< 0.001
	Mite treatment	*F* = 24.385	1, 384	< 0.001
	Migration route	*F* = 3.337	2, 373	0.037
	*Varroa* level	*F* = 53.124	1, 373	< 0.001
	Stock × Mite treatment	*F* = 17.502	1, 375	< 0.001
	Stock × *Varroa* level	*F* = 18.912	1, 360	< 0.001
	Mite Treatment × *Varroa* level	*F* = 1.042	1, 430	0.308
Fixed coefficient	*Varroa* level	Coefficient = 0.123	373	< 0.001
	Pol-line × *Varroa* level	Coefficient = -0.095	360	< 0.001
Holm-Bonferroni Post Hoc Test	Mite treatment: low–high	*t* = 5.940	378	< 0.001
	Stock: commercial—Pol-line	*t* = 8.106	381	< 0.001
	Migration route: Stationary—Mississippi	*t* = 3.563	382	0.001
	Migration route: California—Mississippi	*t* = 2.603	375	0.019
	Low mite treatment: commercial—pol-line	*t* = 7.477	387	< 0.001
	High mite treatment: commercial—Pol-line	*t* = 1.401	386	0.162
Random effect	Colony ID	*Z* = 9.608	–	< 0.001

Symbols INDICATE factor interactions (×), pairwise comparisons (−), and groupings (:), d.f. are calculated via the Welch–Satterthwaite approximation.

Specifically, when examining the influence of *Varroa*, colonies were significantly more likely to die during the course of the study, if they had higher *Varroa* levels (mean_dead_ = 2.698 MPHB, mean_alive_ = 0.914 MPHB; Fig. 3a,b), were in the low mite treatment group (mean survival_low_ = 0.212, mean survival_high_ = 0.645), were of Commercial stock (mean survival_Commercial_ = 0.138, mean survival_Pol-line_ = 0.666), and were in the Stationary or California, rather than Mississippi migration group (mean survival_Stationary_ = 0.184, mean survival_California_ = 0.315, mean survival_Mississippi_ = 0.541) (Table 1). Stock influenced the effect of mite treatment on survival, as Commercial colonies benefitted from an additional mite treatment to a greater extent than did Pol-line colonies, exemplified by their significantly reduced survival at low (mean survival_Commercial_ = 0.028, mean survival_Pol-line_ = 0.625), but not at high mite treatment levels (mean survival_Commercial_ = 0.559, mean survival_Pol-line_ = 0.724) (Table 1). Stock also influenced the effect of *Varroa* level on survival, as being of Pol-line stock significantly improved the prognosis for any given *Varroa* level (Fig. 3b; Table 1), leading to better survival outcomes across infestation strata. Colony ID had a significant random effect (Table 1), indicating a degree of unexplained inter-colony variability in survival outcomes, as would be expected.

In contrast to *Varroa* levels, there was no significant influence of DWV-A, DWV-B, CBPV, or BQCV titres upon colony survival status in February (Fig. 3c,d; Table 2). Additionally, when analysing the relationship between viral titres and survival, no effect of stock, mite treatment, or migration route was present, as colonies were selected based on an even distribution of stocks and migration routes across survival outcomes, to minimise any confounding effects upon viral dynamics (Table 2). As with the *Varroa* data, colony ID had a significant random effect (Table 2).

**Table 2:** Summary statistics for GLMM assessing the effect of viral titres on colony survival.

Type	Factor	Statistic	d.f	*P*
Fixed effect	Stock	*F* = 1.352	1, 64	0.249
	Mite treatment	*F* = 0.216	1, 67	0.644
	Migration route	*F* = 0.135	1, 56	0.715
	DWV-A titre	*F* = 1.026	1, 66	0.315
	DWV-B titre	*F* = 0.206	1, 85	0.651
	CBPV titre	*F* = 0.516	1, 80	0.475
	BQCV titre	*F* = 3.142	1, 69	0.081
Random effect	Colony ID	*Z* = 4.185	–	< 0.001

d.f. are calculated via the Welch–Satterthwaite approximation.

### Predictive differences in Varroa levels and viral titres

**Figure 4:**
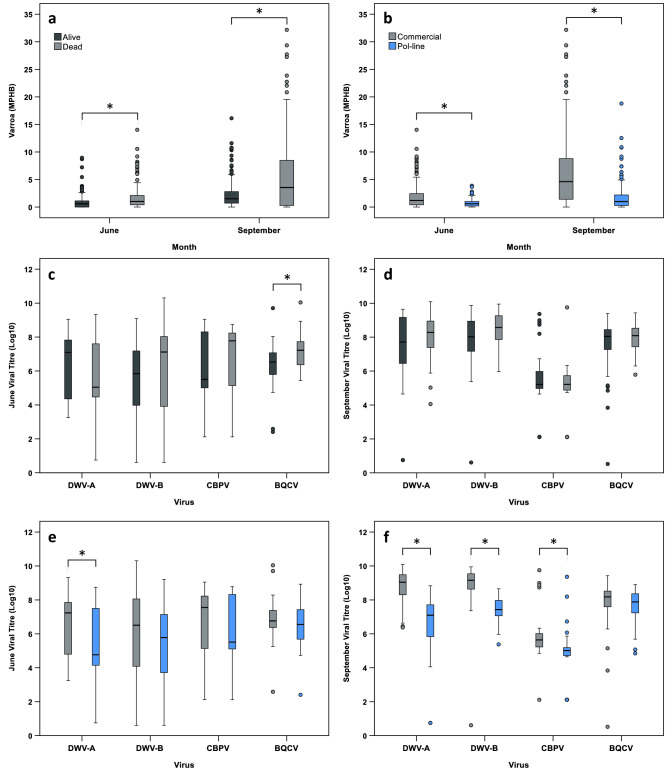
(**a**,**b**) June and September differences in *Varroa* levels (June, *N*_Commercial_ = 190, *N*_Pol-line_ = 167; September, *N*_Commercial_ = 167, *N*_Pol-line_ = 157). (**c**–**f**) June and September differences in viral titres (June, *N*_Commercial_ = 46, *N*_Pol-line_ = 42; September, *N*_Commercial_ = 37, *N*_Pol-line_ = 39). Boxplots are coloured based on survival outcome in February (survived, dark grey; died, light grey) (**a**,**c**,**d**), and stock (Commercial, grey; Pol-line, blue) (**b**,**e**,**f**). Outliers (greater than 1.5 times the interquartile range from the median) are indicated with circles. Asterisks indicate significant differences between groups (η^2^ > 0.060, P < 0.05).

*Varroa* levels were significantly higher in colonies that died during the course of the study, when compared at both June (mean rank_dead_ = 204.520, mean rank_alive_ = 144.590), and September time points (mean rank_dead_ = 201.890, mean rank_alive_ = 119.550) (Fig. 4a; Table 3). Additionally, Pol-line colonies displayed significantly lower *Varroa* levels than Commercial colonies in both June (mean rank_Pol-line_ = 140.850, mean rank_Commercial_ = 212.530) and September (mean rank_Pol-line_ = 106.440, mean rank_Commercial_ = 215.200) (Fig. 4b; Table 3), indicating a predictive association between *Varroa* level, stock, and colony survival.

**Table 3:** Summary statistics for Mann–Whitney *U*-tests comparing predictive differences in *Varroa* levels.

Response	Factor	Statistic	*N*	η^2^	*P*
Survival	June *Varroa* level	*U* = 20,811.000	152, 205	0.082	< 0.001
	September *Varroa* level	*U* = 19,755.000	155, 169	0.193	< 0.001
Stock	June *Varroa* level	*U* = 9,493.500	190, 167	0.120	< 0.001
	September *Varroa* level	*U* = 4,308.000	167, 157	0.337	< 0.001

Eta-squared values indicate factor effect sizes (small, η^2^ ≥ 0.010- < 0.060; intermediate, η^2^ ≥ 0.060- < 0.140; large, η^2^ ≥ 0.140).

Viral titres in June were not significantly different between colonies that died, or survived, for DWV-A (mean rank_dead_ = 40.680, mean rank_alive_ = 46.790), DWV-B (mean rank_dead_ = 49.000, mean rank_alive_ = 41.800), or CBPV (mean rank_dead_ = 45.440, mean rank_alive_ = 43.940) (Fig. 4c; Table 4). However, June BQCV titres were higher in colonies that died by February (mean rank_dead_ = 53.200, mean rank_alive_ = 39.280; Fig. 4c; Table 4). In September, there were no significant differences between colonies that died, or survived, for any of the viral titres measured (DWV-A: mean rank_dead_ = 43.000, mean rank_alive_ = 35.880; DWV-B: mean rank_dead_ = 44.840, mean rank_alive_ = 34.800; CBPV: mean rank_dead_ = 36.960, mean rank_alive_ = 39.400; BQCV: mean rank_dead_ = 41.140, mean rank_alive_ = 36.960; Fig. 4d; Table 4).

**Table 4:** Summary statistics for Mann–Whitney *U*-tests comparing predictive differences in viral titres.

Response	Factor	Statistic	*N*	η^2^	*P*
Survival	June DWV-A titre	*U* = 781.500	55, 33	0.013	0.277
	June DWV-B titre	*U* = 1,056.000	55, 33	0.019	0.200
	June CBPV titre	*U* = 938.500	55, 33	0.001	0.789
	June BQCV titre	*U* = 1,194.500	55, 33	0.070	0.013
	September DWV-A titre	*U* = 798.000	48, 28	0.021	0.175
	September DWV-B titre	*U* = 849.500	48, 28	0.048	0.056
	√-x (September DWV-B titre)	*t* = 1.951	48, 28	0.051	0.055
	September CBPV titre	*U* = 629.000	48, 28	0.003	0.643
	September BQCV titre	*U* = 746.000	48, 28	0.008	0.426
Stock	June DWV-A titre	*U* = 680.500	46, 42	0.065	0.017
	June DWV-B titre	*U* = 753.000	46, 42	0.036	0.075
	June CBPV titre	*U* = 954.500	46, 42	< 0.001	0.923
	June BQCV titre	*U* = 780.500	46, 42	0.027	0.121
	September DWV-A titre	*U* = 124.500	37, 39	0.506	< 0.001
	September DWV-B titre	*U* = 131.500	37, 39	0.495	< 0.001
	September CBPV titre	*U* = 300.000	37, 39	0.252	< 0.001
	September BQCV titre	*U* = 597.000	37, 39	0.022	0.196

Eta-squared values indicate factor effect sizes (small, η^2^ ≥ 0.010– < 0.060; intermediate, η^2^ ≥ 0.060– < 0.140; large, η^2^ ≥ 0.140).

In June, Pol-line colonies showed significantly lower titres of DWV-A than did Commercial colonies (mean rank_Pol-line_ = 37.700, mean rank_Commercial_ = 50.710); however, there were no significant differences between stocks in DWV-B (mean rank_Pol-line_ = 39.430, mean rank_Commercial_ = 49.130), CBPV (mean rank_Pol-line_ = 44.230, mean rank_Commercial_ = 44.750), or BQCV titres (mean rank_Pol-line_ = 40.080, mean rank_Commercial_ = 48.530) (Fig. 4e; Table 4). In September, viral titres were significantly lower in Pol-line colonies for DWV-A (mean rank_Pol-line_ = 23.190, mean rank_Commercial_ = 54.640), DWV-B (mean rank_Pol-line_ = 23.370, mean rank_Commercial_ = 54.450), and CBPV (mean rank_Pol-line_ = 27.690, mean rank_Commercial_ = 49.890) (Fig. 4f; Table 4), as would be expected based on their correlation with *Varroa* levels (Fig. S1a–c). In contrast, however, September BQCV titres did not differ significantly between stocks (mean rank_Pol-line_ = 35.310, mean rank_Commercial_ = 41.860; Fig. 4f; Table 4), and showed no correlation with *Varroa* levels (Fig. S1d).

### Prognostic power of Varroa levels and viral titres

**Figure 5:**
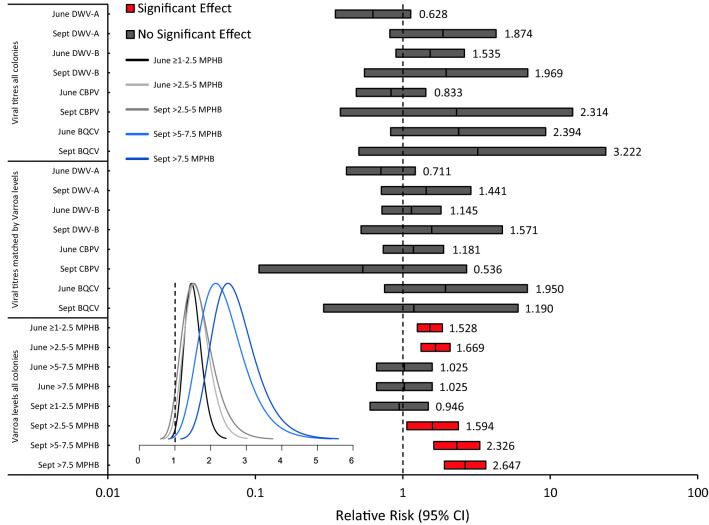
Relative mortality risks of *Varroa* levels (*N*_Commercial_ = 193, *N*_Pol-line_ = 173), viral titres (*N*_Commercial_ = 46, *N*_Pol-line_ = 42), and viral titres controlled for *Varroa* levels (*N*_Commercial_ = 30, *N*_Pol-line_ = 30), as epidemiological exposure factors. Bars display relative risk point estimates, bounded by 95% confidence intervals, displayed on a log_10_ scale to accurately represent proportional change in risk. Colours indicate factor significance (RR, point estimate ± CI_95%_ > 1.00, P < 0.05) as a predictor of mortality (significant effect, red; no significant effect, dark grey). Numbers provide exact relative risk point estimates. The inset displays posterior risk probability densities for significant factors, encompassing 100% credible intervals. Colours indicate factor (June ≥ 1–2.5 MPHB, black; June > 2.5–5 MPHB, light grey; September > 2.5–5 MPHB, dark grey; September > 5–7.5 MPHB, light blue; September > 7.5 MPHB, dark blue). For full details of exposure groupings, see “Materials and methods”.

In June, *Varroa* levels significantly increased the relative risk of mortality by February if colonies fell into the ≥ 1–2.5 (RR, point estimate = 1.528, CI_95%_ 1.256–1.859, AR = 0.238, *P* < 0.001; Fig. 5), or > 2.5–5 (RR, point estimate = 1.669, CI_95%_ 1.330–2.059, AR = 0.303, *P* < 0.001; Fig. 5), MPHB infestation strata. However, the paucity of colonies with *Varroa* levels > 5 MPHB precluded significant prediction of mortality risk above this level (Fig. 5). In September, the initial risk threshold was higher, as *Varroa* levels began to significantly increase the relative risk of mortality only at > 2.5–5 (RR, point estimate = 1.594, CI_95%_ 1.066–2.384, AR = 0.202, *P* = 0.024; Fig. 5), with the risk becoming sequentially greater at > 5–7.5 (RR, point estimate = 2.326, CI_95%_ 1.624–3.333, AR = 0.431, *P* < 0.001; Fig. 5), and > 7.5 MPHB (RR, point estimate = 2.647, CI_95%_ 1.916–3.658, AR = 0.536, *P* < 0.001; Fig. 5). Notably, no other factor significantly influenced the relative risk of mortality. For a list of all factors and risk probability distributions, see (Fig. 5).

### Varroa model

**Figure 6:**
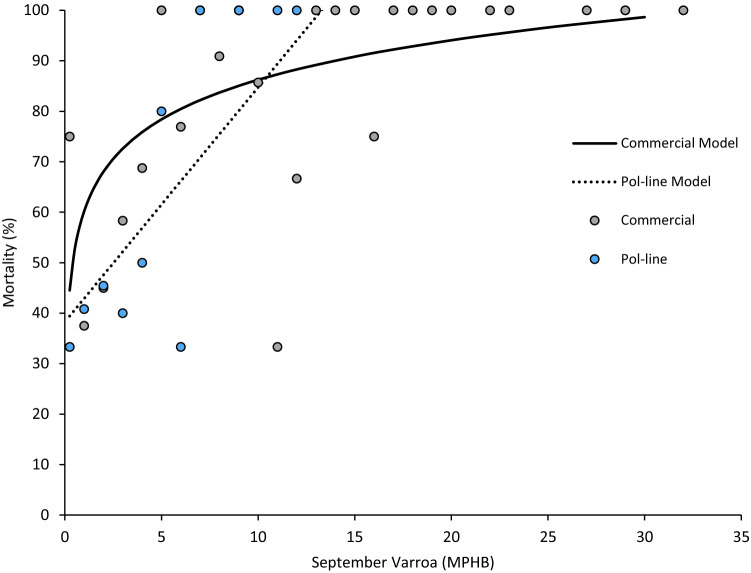
Predictive relationship between September *Varroa* levels and colony mortality (*N*_Commercial_ = 167, *N*_Pol-line_ = 157). Points are based on binned data from all colonies falling into a *Varroa* stratum, and concordant percentage mortality. Colours (Commercial, grey; Pol-line, blue), and line types (Commercial, solid; Pol-line, dashed), indicate stock. Lines are fit against the binned data for each stock (Commercial, *R*^2^ = 0.349, Pol-line, *R*^2^ = 0.647).

The relationship between *Varroa* level in September, and mortality, was explained by a logarithmic function in Commercial colonies (Curve estimation test, *F*_1,24_ = 12.851, *R*^2^ = 0.349, *P* < 0.001; Fig. 6), and a linear function in Pol-line colonies (Curve estimation test, *F*_1,10_ = 18.311, *R*^2^ = 0.647, *P* = 0.002; Fig. 6). At a threshold of 3 MPHB, high mortality was predicted for both Commercial (Curve estimation test, percentage mortality_Commercial_ = 72.618%; Fig. 6), and Pol-line colonies (Curve estimation test, percentage mortality_Pol-line_ = 52.210%; Fig. 6), although this was substantially lower in the latter case. Interestingly, the two functions intersected at 10 MPHB, as despite considerable mortality, some commercial colonies were able to survive with very high infestation levels, as reflected in the reduced explanatory power of the commercial model (Fig. 6).

## Discussion

Our results show that under migratory beekeeping conditions, Pol-line honey bees have significantly enhanced colony and queen survival when compared to a standard commercial Italian stock (Fig. 2a). Additionally, the productivity of Pol-line colonies appears equivalent to that of Commercial honey bees, as evidenced by comparable honey production and population sizes among surviving colonies (Fig. 2b,c). This improved performance is associated with lower *Varroa* levels (Figs. 3b, 4b), and concomitantly reduced titres of DWV-A, DWV-B, and CBPV in September (Fig. 3d, 4f). Notably, the multiplicative effect of *Varroa* levels on colony survival is significantly weaker in Pol-line colonies, leading to better survival prognoses at all but the highest infestation strata (Figs. 3b, 6; Table 1). An ostensible explanation for this is the constant removal of infested brood by Pol-line workers, as is characteristic of VSH behaviour^48,54^, and thus a subsequent dampening of *Varroa* growth dynamics and viral transmission. Consequently, Pol-line colonies that received only one mite treatment demonstrated survival rates empirically equivalent to Commercial colonies receiving two. Indeed, the benefit of additional mite treatments was generally greater for Commercial colonies, as the majority of Pol-line colonies naturally maintained *Varroa* levels below the recommended treatment threshold^114^. This has considerable implications both for improving colony survival, and reducing the escalating need for acaricide use^27^.

To our knowledge, these data constitute the fullest characterisation of improved functionality in a *Varroa*-resistant honey bee stock to date. Further, as the queens used in this study were mated via drone saturation, and the resultant colonies retained genetics for mite-resistance, our results are directly applicable to industrial implementation^128^. It should be noted that the present study comprises only a single year of data, and thus further work will be needed to validate Pol-line derivatives prior to commercial introduction. Encouragingly however, commercial field trials of untreated ‘Hilo’ bees^60^—a stock derived from Pol-line beginning in 2015—have shown superior overwinter survival, although slightly lower honey production, when compared to acaricide-treated commercial Italian, and Italian × Carniolan colonies (RGD, personal communication)^129^. Notably, allele frequency analyses place Pol-line and Hilo bees in a distinct genetic cluster, significantly differentiated from other commercial and research stocks currently used in the United States^60^. As such, our results serve as a promising empirical characterisation of the potential for derived *Varroa*-resistant stocks to fundamentally improve modern beekeeping.

The comparatively high mortality rates observed in this study^7,8^ are a consequence of two experimental design attributes. In order to assess the true resilience of colonies to *Varroa*, we necessarily included a minimal mite treatment group for each stock, resulting in significantly lower overall acaricide use than is standard in commercial operations. As such, *Varroa* levels were high, and its presence pervasive, with concordant implications for mite spillover and mortality prior to the second acaricide treatment—especially in the Commercial colonies. Additionally, industrial pollination operations place varied stresses on colonies, due to intensive transit, climatic extremes, high colony densities, and fluctuating local conditions^57,63,130^. The increased mortality levels observed in the stationary migration group exemplify this stochasticity, as colonies experienced a starvation event during the winter, leading to severe losses (Table 1). Such a result serves to highlight the importance of management regime and local stressors in influencing colony outcomes^130^, and is pertinent when considering distributed pollination systems. Notably, our results are not inconsistent with other commercial scenarios in which *Varroa* pressure is high, which is very often the case^82,131^. Accordingly, the present study constitutes a controlled but rigorous test, in a large-scale commercial operation, under significant parasite burden. In the case of stock validation, such a setup is preferable, as we wished to determine the continued efficacy of Pol-line’s outcrossed VSH traits^54^, and substantial *Varroa* pressure was required to do so effectively.

The findings from our assessment of *Varroa*, DWV-A, DWV-B, CBPV, and BQCV, and their influences upon colony mortality, were notable. In agreement with previous studies, *Varroa* levels were the single strongest predictor of colony mortality for both stocks, diverging as early as June, and peaking in September (Figs. 3, 4a,b; Tables 1, 3). Interestingly, the impacts of both early season and pre-winter *Varroa* levels on subsequent mortality, suggest that current treatment thresholds may not be conservative enough to curtail substantial losses (Figs. 3a, 6). Indeed, additional probit models support this assertion for the present data (Fig. S2). In contrast, titres of DWV-A, DWV-B, CBPV, and BQCV were not significant independent determinants of colony survival (Figs. 3c,d, 4c; Table 2). The only significant viral difference was in the case of June BQCV titres, which were higher in colonies that went on to die, however the effect size was intermediate (Table 4), and did not carry over into multivariate analyses, indicating a lack of causality.

While September titres of DWV-A, DWV-B, and BQCV were on average numerically greater in colonies that died, this difference was not significant when controlling for covariates, or when taken as a predictive measure, and levels of CBPV were in fact lower in colonies that were later lost (Figs. 3c, 4d; Table 2). DWV-A and DWV-B demonstrated broadly similar patterns across survival outcomes and stocks, except in December, in which DWV-B dropped to a greater extent in Commercial colonies than did DWV-A (Fig. 3d). The titres of DWV-A, DWV-B, and CBPV were significantly reduced in Pol-line colonies during September, and in the case of DWV-A, also during June; however BQCV showed no differences between stocks (Fig. 4e,f). This may be explained by the lower *Varroa* levels present in Pol-line colonies, with the greatest differences emerging in September (Figs. 3b, 4b; Table 3), and hence concomitantly reduced levels of directly and indirectly *Varroa*-associated pathogens (Figs. S1a–c). Notably, such a trend indicates that BQCV did not exhibit significant *Varroa* association, and is corroborated by its lack of direct correlation with September *Varroa* levels (Fig. S1d). This finding is pertinent to the etiological understanding of BQCV, as it suggests that despite potential replication in the mites^102^, and temporal correlations with *Varroa* invasion^22^, there is poor empirical evidence for an epidemiologically meaningful transmission pathway.

In contrast to DWV-A and DWV-B, the *Varroa* association of CBPV was comparatively weak (Fig. S1c). It is thus possible that stock-specific viral resistance mechanisms^132,133^, or destabilised immune function as a result of *Varroa* burdens^134^, played a role in the differences observed between stocks (Fig. 4f). Host–pathogen genotypic interactions are of interest in honey bee stock selection^38^, and may warrant further investigation in both Pol-line bees, and other mite-resistant varieties^66,94^. However, it will be important to separate such effects from pathogen opportunism, especially when stocks confer broadly improved colony health.

Taken together, our data suggest that DWV-A, DWV-B, and to a lesser extent CBPV titres, track *Varroa* levels, but are comparatively weaker predictors of colony survival outcome than *Varroa* itself. Relative and attributable risk analyses provided further insight into these results. When only colonies grouped by comparable *Varroa* levels were examined, differing titres of DWW-A, DWV-B, CBPV, and BQCV never constituted significant additive predictors of mortality risk (Fig. 5). Further, the only relative risk measures with a significant effect on mortality, belonged to those of *Varroa* levels alone (Fig. 5).

These findings are relevant to the understanding of DWV-A and DWV-B as causative agents of colony loss. Our data suggest that *Varroa*, as a factor, consistently has the greatest influence on mortality, likely as it encompasses both the effects of mechanical damage, via feeding, and the transmission of associated pathogens. While DWV and other viruses correlate with colony mortality, and have been shown both to kill, and sub-lethally influence bees^65,135^, it appears that when these effects are decoupled from their correlation with *Varroa* levels, their predictive power is significantly reduced. The crucial methodological and diagnostic implication of this, is that, at a minimum, compared to the viruses tested here, *Varroa* has significant additive power in predicting colony outcome. This additive effect may be explained by damage to the host’s fat body^12,21^, or additional latent pathogen transmission unaccounted for by single target measures^17^, and is corroborated by previous experimental evidence^76,83,101,136^. While such a trend does not dismiss the importance of *Varroa*-vectored viruses, it does suggest that the harm caused by *Varroa* feeding itself has been chronically underestimated, and implies that the established paradigm of *Varroa*-mediated colony loss may be incorrect. Notably, the comparatively diminished effect of viruses observed in our study, can be explained by the simultaneous use of a controlled mixed modelling approach, a commercially realistic sample size, and measures of viral titre only—rather than viral prevalence. The latter aspect is salient, as the potentially latent etiology of DWV infections^137^ necessitates a linkage between actual viral titres and mortality, especially if causative associations are to be made. It is true that *Varroa*-vectored viruses correlate with *Varroa* infestation levels, but the relationship is not monotonic^1,22^, and thus future studies linking these pathogens to mortality should include or control for *Varroa* levels, if they are to produce meaningful insight.

When considering colony losses, the relative weightings of DWV, or indeed any of the viruses vectored by *Varroa*, have never been effectively isolated from the effect of *Varroa* feeding *per se*^12^. It should be noted that, as demonstrated here, other datasets have found variable causative influences of viral titres upon bee health^138^, and colony mortality^81,136^. Further, to date, no studies have been able to decouple *Varroa*-virus mortality correlations from *Varroa* mortality correlations at the colony-level. Based on this, it is somewhat unclear as to why vectored viruses are assumed to be the prime, if not sole, causative agents of *Varroa*-mediated colony losses^38,139^, especially as the parasite’s mode of feeding necessitates organ damage^12^. Indeed, in certain cases, *Varroa* levels alone predict mortality, while viral titres fail to do so^83,136^. It is well established that by removing or controlling for *Varroa* mites, associated viruses may be managed indirectly^111,112^, thus, there is practical merit in focussing on *Varroa*, it being the upstream epidemiological factor. This is well-illustrated by the finding that while VSH behaviour can in fact lead to the spread of DWV via pupal cannibalism in the lab^140^, it nevertheless reduces DWV titres at the colony-level in the field (Figs. 3d, 4f), as it mitigates a more potent transmission pathway—that of *Varroa* feeding. In tandem with recent advances in the understanding of *Varroa* etiology^12^, a growing body of research also indicates that the mites themselves are not harmless vectors, but invasive epizootic parasites, that seriously damage their naïve hosts via destructive lipophagy. Alongside the present findings, this warrants serious revaluation of the relative importance of *Varroa*, and its transmitted viruses, in causing colony-level harm. Multifactorial approaches to understanding *Varroa*-virus dynamics, especially in relation to factor weightings, will be important in future epidemiological studies, as arguably, *Varroa*-mediated colony mortality still requires substantial mechanistic elucidation.

Our results demonstrate that *Varroa*-resistant honey bees present strong potential for reducing colony losses in commercial beekeeping operations. Incorporating the VSH trait into commercial stocks appears to engender both *Varroa*-resistance, and favourable colony productivity. Furthermore, we find that DWV-A, DWV-B, CBPV, and BQCV titres are inferior predictors of colony mortality when compared to *Varroa* levels alone. This implies that the relative damage caused by *Varroa* feeding per se, and the pathogens transmitted by this process, will require further investigation if effective mitigation methods are to be developed. In sum, our data suggest that a *Varroa*-centric control strategy, incorporating the adoption of resistant stocks, currently represents the most effective, sustainable, and technologically tractable solution to global honey bee losses.

## Supplementary Information

Supplementary Figures.

Supplementary Tables.

## Data Availability

The authors declare that all supporting data is available within the supplementary information.
