# Supplementary material for: A derived honey bee stock confers resistance to Varroa destructor and associated viral transmission
Source: Sci Rep. 2022 Apr 7;12:4852. doi: 10.1038/s41598-022-08643-w (PMC8989980; doi:10.1038/s41598-022-08643-w)
Supplement: Supplementary file 1 — Supplementary Figures. [file 41598_2022_8643_MOESM1_ESM.pdf]

A Derived Honey Bee Stock Confers Resistance to *Varroa destructor* and Associated Viral Transmission

Thomas A. O'Shea-Wheller, Frank D. Rinkevich, Robert G. Danka, Michael Simone-Finstrom, Philip G. Tokarz, Kristen B. Healy

Supplementary Information

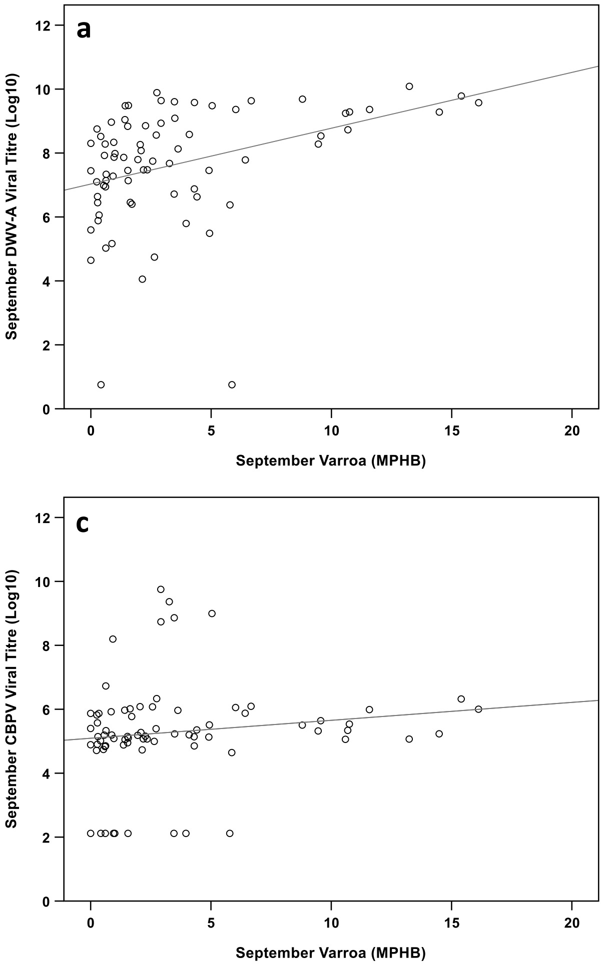

Fig. S1. Correlations between *Varroa* levels and titres of DWV-A (Spearman's rank correlation, *Varroa*/DWV-A: ρ=0.459, N=76, P<0.001) (R^2=0.142) (a), DWV-B (Spearman's rank correlation, *Varroa*/DWV-B: ρ=0.419, N=76, P<0.001) (R^2=0.123) (b), CBPV (Spearman's rank correlation, *Varroa*/CBPV: ρ=0.278, N=76, P=0.015) (R^2=0.019) (c), and BQCV (Spearman's rank correlation, *Varroa*/BQCV: ρ=0.187, N=76, P=0.105) (R^2=0.014) (d), in colonies during September.

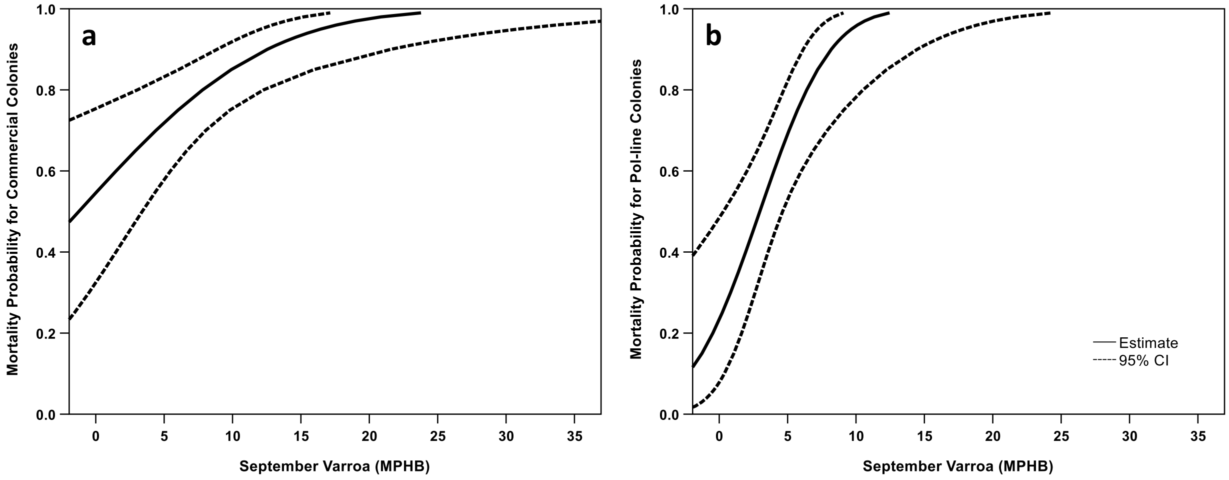

Fig. S2. Projected relationship between September *Varroa* levels and colony mortality, in Commercial (Probit equation, Y=0.24+0.03(*Varroa*)) (R^2=0.071) (Probit model, effect of *Varroa*: χ^2=16.477, d.f.=1, P<0.001) (a), and Pol-line (Probit equation, Y=0.34+0.08(*Varroa*)) (R^2=0.154) (Probit model, effect of *Varroa*: χ^2=15,524, d.f.=1, P<0.001) (b) colonies ($N_{\text{Commercial}}$=167, $N_{\text{Pol-line}}$=157). Lines indicate dose-response probit curves (solid), and corresponding 95% confidence intervals (dashed).

Table S1. Summary statistics for Mann–Whitney *U*-tests comparing initial frame-counts and *Varroa* levels between stocks.

**Response**	**Factor**	**Statistic**	***N***	**η^2**	***P***
**Stock**	May Frame-Count	*U*=15,225.000	193,173	0.002	0.191
	May *Varroa* Level	*U*=15,070.000	193,173	0.001	0.513

Eta-squared values indicate factor effect sizes (small, $\eta^2 \geq 0.010$-<0.060; intermediate, $\eta^2 \geq 0.060$-<0.140; large, $\eta^2 \geq 0.140$).

Table S2. Primer sequence information.

Target	Forward sequence	Reverse sequence	Reference	Linearity (r^2)	Reaction efficiency (E)
**DWV-A**	GAG ATT GAA GCG CAT GAA CA	TGA ATT CAG TGT CGC CCA TA	[1]	0.997	103.00%
**DWV-B**	CTG TAG TTA AGC GGT TAT TAG AA	GGT GCTTCT GGA ACA GCG GAA	[2]	0.987	92.95%
**CBPV**	CGC AAG TAC GCC TTG ATA AAG AAC	ACT ACT AGA AAC TCG TCG CTT CG	[3]	0.998	101.30%
**BQCV**	TTT AGA GCG AAT TCG GAA ACA	GGC GTA CCG ATA AAG ATG GA	[4]	0.993	102.94%

References

1. Boncristiani, H. *et al.* Direct effect of acaricides on pathogen loads and gene expression levels in honey bees Apis mellifera. *J. Insect Physiol.* **58**, 613–620 (2012).

2. Ryabov, E. V. *et al.* Recent spread of Varroa destructor virus-1, a honey bee pathogen, in the United States. *Sci. Rep.* **7**, 1–10 (2017).

3. Blanchard, P. *et al.* Evaluation of a real-time two-step RT-PCR assay for quantitation of Chronic bee paralysis virus (CBPV) genome in experimentally-infected bee tissues and in life stages of a symptomatic colony. *J. Virol. Methods* **141**, 7–13 (2007).

4. Simone-Finstrom, M., Aronstein, K., Goblirsch, M., Rinkevich, F. & de Guzman, L. Gamma irradiation inactivates honey bee fungal, microsporidian, and viral pathogens and parasites. *J. Invertebr. Pathol.* **153**, 57–64 (2018).

Table S3. Thermal Protocols.

Target	Taq DNA polymerase activation step	Thermal protocol
**DWV-A**	5 min at 95°C	40 cycles of 5 s at 95°C, 10 s at 53.5°C, and 10 s at 72°C
**DWV-B**	5 min at 95°C	40 cycles of 5 s at 95°C, 10 s at 59°C, and 10 s at 72°C
**CBPV**	5 min at 95°C	40 cycles of 5 s at 95°C, 10 s at 53.5°C, and 10 s at 72°C
**BQCV**	5 min at 95°C	40 cycles of 5 s at 95°C, 10 s at 59°C, and 10 s at 72°C
